# A rare case of pseudoaneurysm caused by *Listeria monocytogenes*

**DOI:** 10.3389/fmed.2025.1615160

**Published:** 2025-09-22

**Authors:** Dee Xi, Chunhua Luo, Qianyuan Li, Shaohua Xu, Xingrong She, Yan Gao

**Affiliations:** ^1^Department of Clinical Laboratory, The First College of Clinical Medical Science, China Three Gorges University, Yichang Central People’s Hospital, Yichang, China; ^2^Department of Emergency, The First College of Clinical Medical Science, China Three Gorges University, Yichang Central People’s Hospital, Yichang, China; ^3^Department of Oncology, The First College of Clinical Medical Science, China Three Gorges University, Yichang Central People’s Hospital, Yichang, China

**Keywords:** *Listeria monocytogenes*, mycotic aneurysm, pseudoaneurysm, brachial artery, blood culture

## Abstract

Mycotic aneurysm is rare in clinical practice, but it is dangerous because it may expand rapidly, rupture, and threaten the patient’s life if left untreated. The common pathogens include *Staphylococcus*, *Salmonella*, and *Streptococcus* species. Infection caused by *Listeria monocytogenes* is very rare, with no more than 40 cases reported worldwide to date. To our knowledge, this is the first case of a pseudoaneurysm in the brachial artery caused by *L. monocytogenes*. Most previously reported cases involved a single aneurysm; however, the case we report is very different. The patient had a medical history of hypertension and cerebral infarction and suffered from aneurysms and pseudoaneurysms at different sites on more than one occasion. The patient was admitted to our hospital due to a pseudoaneurysm in the brachial artery of the left upper limb. Blood culture suggested the infection was caused by *L. monocytogenes*. The inflammation was almost controlled after 1 month of antibiotic therapy, after which surgery was performed to remove the pseudoaneurysm and reconstruct the left brachial artery using an autologous great saphenous vein graft. Postoperatively, the patient continued to receive antibiotics and was discharged 1 week later in good condition.

## 1 Introduction

Mycotic aneurysms are very rare in clinical practice, accounting for only 0.5%–1.53% of all aortic aneurysms in Western countries and reportedly higher in East Asia (1–3). They usually occur in large arteries, such as abdominal aorta, the femoral arteries (4, 5), iliac artery and internal carotid artery (6). The most common pathogens are *Staphylococcus, Salmonella, and Streptococcus* species (4, 7–10). However, infection caused by *Listeria monocytogenes* is extremely rare, with fewer than 40 cases reported to date. Here, we present a rare case of a pseudoaneurysm in the brachial artery caused by *L. monocytogenes*.

## 2 Case presentation

A 46-year-old man was admitted to our hospital with 1 week of continuous pain in his left forearm without any known cause. The pain did not improve with rest. He did not report dizziness, chest pain, abnormal limb movement or sensation, cough, or other discomfort. His medical history included hypertension, cerebral infarction, aortic replacement surgery (time unknown) for aortic dissection at another hospital, surgical intervention for a deep femoral aneurysm in the right lower limb in 2019, and resection of a mesenteric pseudoaneurysm with autologous great saphenous vein reconstruction in 2020.

On admission, physical examination revealed a blood pressure of 145/82 mmHg, heart rate of 86 beats/min, body temperature of 38.2°C, and respiratory rate of 18 breaths/min. Both the upper and lower limbs were edematous, and the left forearm was tender. The color and temperature of the skin of the left upper limb were normal, but the arterial pulse was absent. In the lower extremities, skin temperature was normal, scattered skin lesions were observed, and some areas were slightly red. The dorsal foot arterial pulse was absent.

**FIGURE 1:**
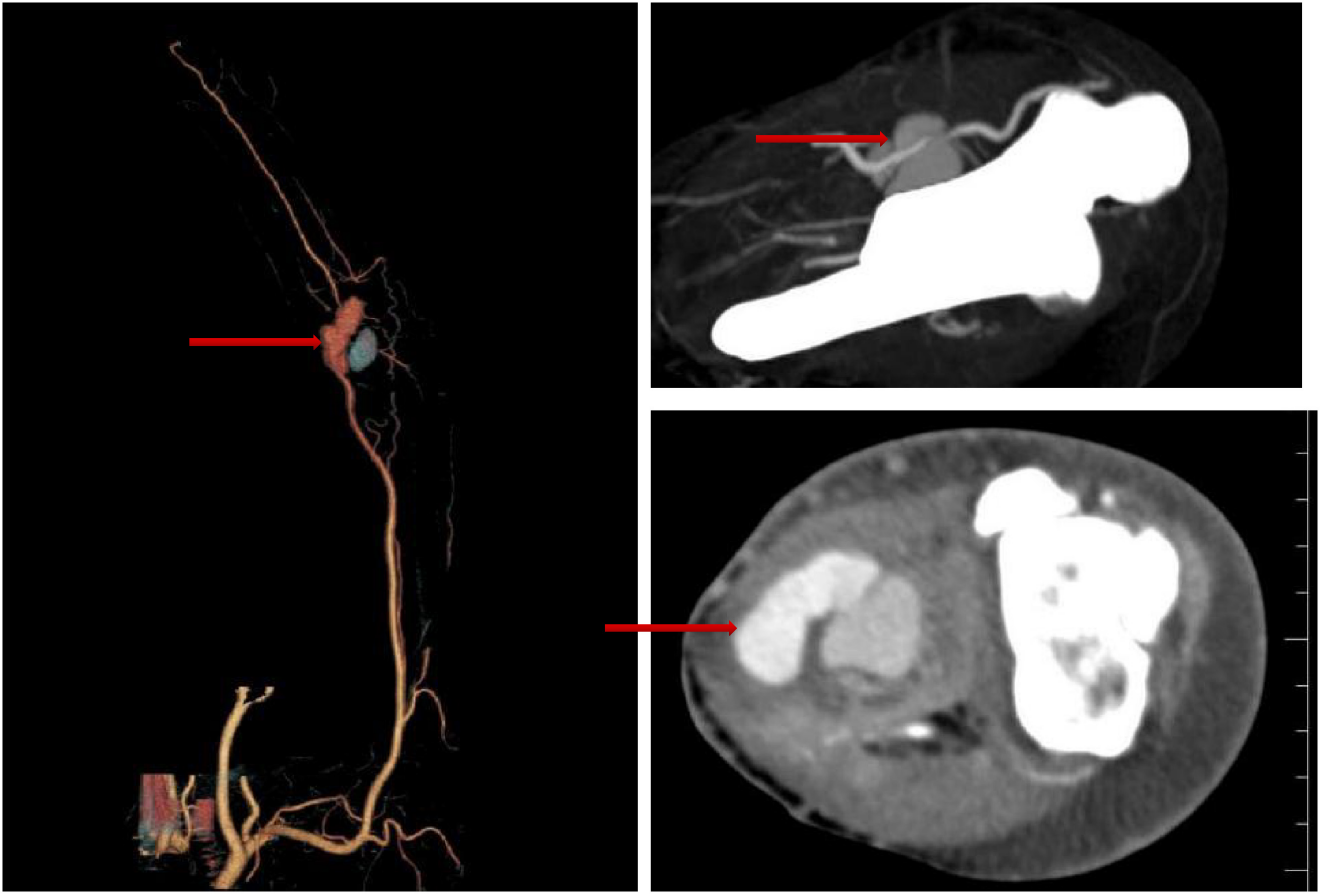
CTA showing the pseudoaneurysm in the patient’s left distal brachial artery (red arrows indicate the lesion).

Blood tests revealed a procalcitonin level of 0.43 ng/mL, C-reactive protein of 125.75 mg/L, erythrocyte sedimentation rate of 34 mm/h, IL-6 of 55.27 pg/mL, and neutrophil percentage of 77.7%. Color Doppler ultrasound of the bilateral lower limb veins showed thrombosis in the left tibioperoneal artery trunk. Computed tomography angiography (CTA) showed a pseudoaneurysm in the left distal brachial artery (Figure 1).

Microscopic examination of the skin nodules in the right lower limb revealed a pustule under the cuticle and many acute and chronic inflammatory cells around small blood vessels in the dermis and skin appendages. Immunohistochemical analysis of the skin nodules showed IgG and IgG_4_ were negative, CD38 was scatteredly positive, CD138 was negative, and Ki-67 was scatteredly positive.

The patient was initially treated empirically with piperacillin-tazobactam, but the therapeutic effect was unsatisfactory, and fever persisted. Two sets of aerobic and anaerobic blood cultures (BacT/Alert FAN plus, bioMérieux, Marcy I’Etoile, France) were obtained. One anaerobic bottle of blood cultures turned positive after about 22 h and the other three bottles turned positive after about 23.3 h. Gram staining of blood smear showed Gram-positive bacilli (Figure 3a). The result was reported to the clinician immediately. The blood cultures were transferred to blood plates and chocolate plates, which were placed in the incubator at 5% CO_2_, 35 °C. Small grayish white colonies with a diameter of about 1.5 mm can be seen on the plates 24 h later (Figures 3b, c). Narrow β -hemolytic rings can be seen when the colonies on the blood plate were scraped with an inoculating loop. Gram-positive bacillus can be seen via Gram staining of the colonies (Figure 3d), which were identified as *L. monocytogenes* by mass spectrometry (Vitek MS, BioMérieux, France).

A multidisciplinary discussion was conducted to search for the etiology, but no clear cause was found except for the advice to treat the infection based on the blood culture results. We considered whether the pseudoaneurysm was associated with *L. monocytogenes*. Literature review revealed only a few reported cases of *L. monocytogenes* causing aneurysms. After communicating with the patient’s attending doctor, the therapy was adjusted. The antibiotic regimen was changed to ampicillin plus amikacin. Following this, the patient’s procalcitonin levels and other inflammatory markers gradually decreased, and his body temperature normalized.

One month later, the patient underwent resection of the pseudoaneurysm in his left brachial artery and reconstruction with an autologous saphenous vein graft. Postoperative antibiotic therapy continued until the procalcitonin level normalized. When the patient’s condition improved, he was discharged a few days later.

**FIGURE 2:**
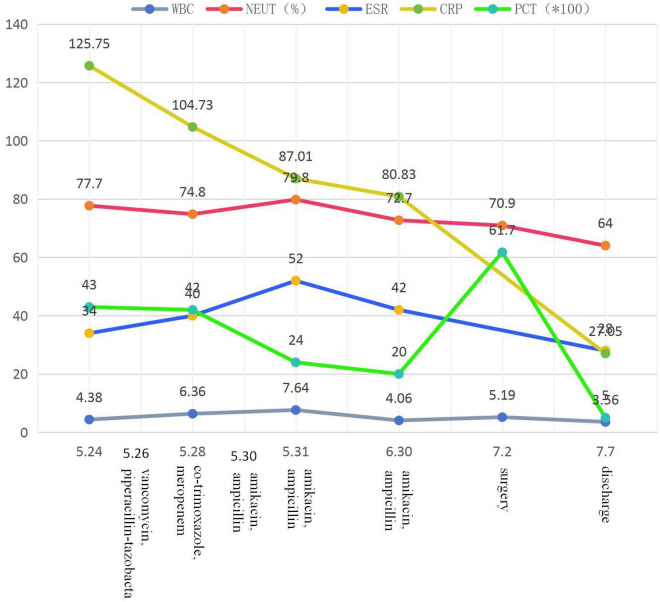
The change of inflammatory indicators.

The changes in the patient’s inflammatory indicators during treatment are shown in Figure 2 and Table 1.

**TABLE 1:** Changes in inflammatory indicators during treatment.

Date	PCT (ng/mL)	WBC (*10^9^/L)	NEUT (%)	ESR (mm/h)	CRP (mg/L)	Treatment
2021-05-24	0.43	4.38	77.7	34	125.75	Vancomycin, piperacillin-tazobactam
2021-05-28	0.42	6.36	74.8	40	104.73	Trimethoprim-sulfamethoxazole, meropenem
2021-05-30						Amikacin, ampicillin
2021-05-31	0.24	7.64	79.8	52	87.01	Amikacin, ampicillin
2021-06-30	0.20	4.06	72.7	42	80.83	Amikacin, ampicillin
2021-07-02	0.617	5.19	70.9			Surgery
2021-07-07	0.05	3.56	64.0	28	27.05	Discharge

**FIGURE 3:**
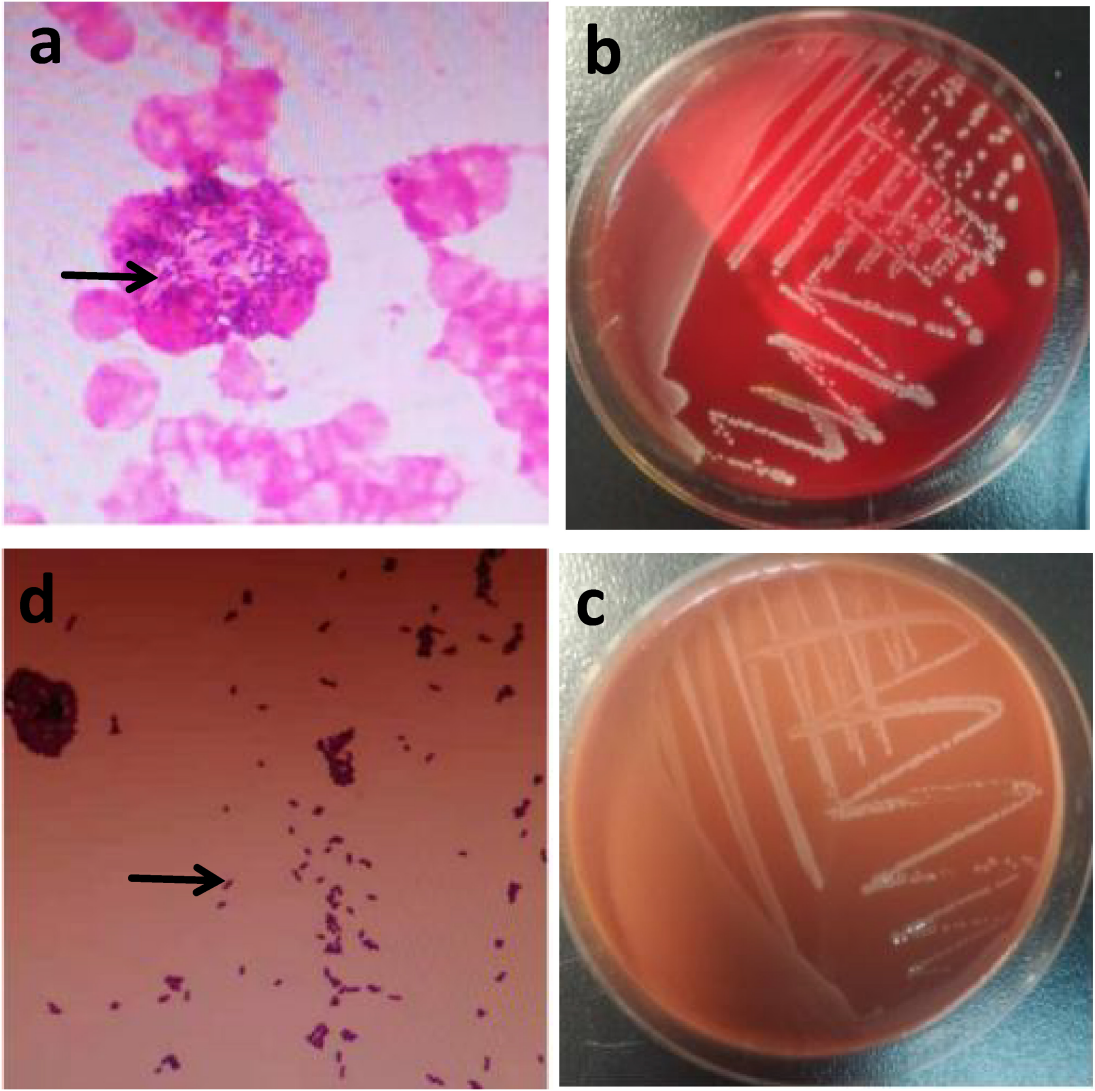
Gramstaining and colonies characters of *Listeria monocytogenes*. Gram staining of blood smear showed Gram-positive bacilli (the arrow shows) **(a).** The blood plate **(b)** and the chocolate plate **(c)** grew small grayish white colonies with a diameter of about 1.5 mm after 24 h of incubation in the incubator at 35°C in: 5% CO_2_. Gram staining of colonies showed Gram-positive bacilli (the arrow shows) **(d).**

## 3 Discussion

*Listeria monocytogenes* is a Gram-positive, non-sporulating, facultatively anaerobic bacillus (4, 10, 11). It is widely distributed in nature and can be found in soil, rivers, and the bodies of insects, fish, and other organisms (8). Infection through food consumption is the most common route of human infection with *L. monocytogenes*. Humans are usually infected by consuming contaminated milk or meat. Notably, this bacterium can survive at low temperatures (4 °C) (4), so eating food stored in the refrigerator may also lead to infection.

In immunocompromised patients, *L. monocytogenes* typically causes prolonged low-level bacteremia. In immunocompetent individuals, it can survive and replicate inside macrophages, usually causing self-limiting gastroenteritis (4, 12–14). High-risk groups include infants, pregnant women, the elderly, and immunocompromised individuals (4, 5, 15); however, in rare cases, invasive disease can occur in immunocompetent adults and children (16). Clinical manifestations include nausea, vomiting, abdominal pain, diarrhea, fever, headache, meningitis, sepsis, endocarditis, meningoencephalitis, hepatapostema, encephalopyosis, cholecystitis, cholecystitis, arthritis, osteomyelitis (9, 15). Infection during pregnancy can lead to abortion. Therefore, at-risk individuals should fully heat refrigerated foods before consumption to prevent infection.

Two cases of farmers who processed and consumed raw milk and dairy products infected by *L. monocytogenes* had been reported, including cases of periaortic endograft infection (17) and abdominal aortic aneurysm (18). Maybe occupational risk factors should be considered in clinical practice. However, in this case we report the patient did not have these risk factors of exposure to *L. monocytogenes*.

The term “mycotic aneurysm” was firstly proposed by William Osler in 1885 on a lecture, referring to the fungal-like vegetations associated with infected aneurysms in endocarditis (19). Since then, numerous cases of infective aneurysms caused by different pathogens have been reported. “Mycotic aneurysm” is also known today as “infective native aortic aneurysm” (20).

Currently, bacteria are the most common cause of mycotic aneurysms. In Europe and North America, *Staphylococcus* species are the leading pathogens, followed by *Streptococcus* and *Enterobacteriaceae* such as *Escherichia coli* and *Salmonella*. In East Asia, *Salmonella* species are the most common pathogens (21). Infection caused by *L. monocytogenes* remains very rare, with no more than 40 cases reported worldwide.

Typically, the normal intimal layer protects artery walls from infection. However, when the intimal layer is damaged–due to conditions such as atherosclerosis, degenerative aneurysms, non-infectious arteritis, or congenital anomalies–the resistance is weakened, making arteries more susceptible to infection (7, 12, 22).

Several mechanisms may explain arterial infection by *L. monocytogenes*. After ingestion of contaminated food, the bacteria can penetrate the intestinal mucosa and enter the bloodstream, subsequently adhering to the endocardium or arterial walls. *L. monocytogenes* can then spread from cell to cell and invade local aortic tissue (13). Enteric infections following aortic graft repairs can also spread to the arterial wall, causing graft displacement, aortic dilatation, and rupture (22). Mycotic aneurysms caused by *L. monocytogenes* are rare and often involve large arteries, frequently in association with prosthetic material (23).

Patients with mycotic aneurysms usually present with fever and pain, or neurological deficits, as seen in cases of ischemic strokes linked to carotid aneurysms (6). However, mycotic aneurysms caused by *L. monocytogenes* may sometimes lack fever (8, 12–14) or pain (9), which necessitates careful clinical examination. Most patients present with leukocytosis, although exceptions exist (4).

Diagnosis often requires imaging studies and microbiological confirmation through blood cultures or tissue sampling (22). The first choice for confirming mycotic aneurysm is computed tomography angiography (CTA) (24), while other methods such as magnetic resonance computed tomography (MRTA), 18-FDG PET or white blood cell scintigraphy (WBCS) can serve as supplementary diagnostics (21, 22). Identifying the pathogen is a critical step in diagnosis. Mycotic aneurysms caused by *L. monocytogenes* can be diagnosed by blood or tissue culture. However, no source of infection is detected in approximately one-third of patients, and pathogens are not isolated in 21%–40% of cases (22). Approximately 20%–30% of cultures are negative (25). When culture results are negative–particularly for difficult-to-culture bacteria such as anaerobes, or when antibiotics have been administered prior to sample collection–PCR and 16S rRNA sequencing can be employed. Additionally, 18S rRNA sequencing may help identify fungal infections (26). We can also consider other samples such as CSF, meconium, and so on to help diagnose listeria except for mycotic aneurysms caused by *L. monocytogenes*.

Targeted antimicrobial therapy based on etiological results can help avoid antibiotic misuse. In these cases, clinicians should also consider rare pathogens such as *Brucella* and *L. monocytogenes*, which are often overlooked. While the diagnosis of mycotic aneurysm primarily relies on clinical manifestations and CTA, relevant laboratory investigations are also essential. Initial assessments should include inflammatory markers (such as erythrocyte sedimentation rate and C-reactive protein), complete blood count, liver and kidney function tests, and blood cultures to identify underlying risk factors.

Treatment of mycotic aneurysm requires prompt and effective antibiotic therapy combined with timely surgical intervention once a definitive diagnosis is made, as non-operated patients have a poor prognosis (5) and successful surgical management can lead to favorable outcomes, with no recurrence reported in some cases (6). Without treatment, aneurysms will expand and eventually rupture, endangering the patient’s life. Empirical antimicrobial therapy should be started before pathogen identification, with subsequent adjustments based on culture results. The duration of antibiotic therapy varies based on individual circumstances, ranging from 4–6 weeks to lifelong therapy (21).

However, the rarity of these cases necessitates further research to establish standardized treatment protocols and improve patient outcomes (27). Although surgical intervention is generally recommended due to the high rupture risk, there have been cases of successful survival with conservative treatment (7). The mortality rate associated with surgery is significantly lower compared to antibiotic therapy alone (4, 7). Due to the rarity of infectious aneurysms, the optimal timing of surgery remains controversial. Some advocate immediate surgery to prevent rupture, while others recommend initial antibiotic treatment followed by delayed surgery once infection is controlled. Statistics indicate that emergency surgical repair has a higher 30-day mortality rate compared to elective surgery (28). The 2024 ESVS Management Guidelines for Abdominal Aortoiliac Aneurysms recommend early surgery regardless of aneurysm size unless strict surveillance can be maintained, as delayed intervention increases the risk of rupture (21).

Penicillin or broad-spectrum penicillin including ampicillin and amoxicillin are the first-line treatments for *L. monocytogenes* infection, and the combination with aminoglycosides may be a better choice for they can act synergistically (8, 22). Erythromycin, vancomycin, rifampicin, and trimethoprim-sulfamethoxazole are appropriate medications as well (22). Ciprofloxacin can also be considered in treatment (29, 30). However, cephalosporins are ineffective due to the bacterium’s intrinsic resistance (10). For patients with penicillin allergy, trimethoprim-sulfamethoxazole and vancomycin are best alternatives (8), although treatment failures with vancomycin or ciprofloxacin have been reported (10).

In this case, the patient initially received piperacillin-tazobactam and vancomycin empirically; however, the treatment was ineffective. After blood cultures confirmed *L. monocytogenes* infection, the antibiotics were changed to amikacin and ampicillin, leading to resolution of the fever and gradual decrease of inflammatory markers. The patient’s procalcitonin levels increased on the day of surgery, likely due to surgical stress.

In patients younger than 60 years with multiple or atypical vascular aneurysms, a specific diagnostic approach is necessary to screen for underlying genetic or connective tissue disorders (21). Our patient, who was 46 years old and had aneurysms at multiple sites, may have had an underlying genetic predisposition. Unfortunately, genetic screening was not performed before discharge.

## 4 Conclusion

The patient in our case had a medical history of hypertension and atherosclerosis, which are high-risk factors for cardiovascular diseases such as aortic dissection and aortic aneurysm. His vessel walls were susceptible to bacterial infection because the intimal layer may have been damaged due to his atherosclerosis. The patient also had severe anemia and a decreased lymphocyte count, indicating reduced immunity. Therefore, based on these factors, the patient was prone to infection by *Listeria monocytogenes*.

This patient had a history of aortic replacement, and the graft may have been infected by *L. monocytogenes*, which could have spread to the bloodstream, caused bacteremia, and subsequently adhered to other vessel walls, leading to the formation of multiple aneurysms. The patient may have been infected by *L. monocytogenes* during prior surgical procedures to treat the deep femoral aneurysm and mesenteric pseudoaneurysm, but the infection might have been overlooked because he did not exhibit obvious symptoms such as fever.

Therefore, when encountering patients with aneurysms that are difficult to diagnose, uncommon etiologies, such as bacterial infection, should be considered if common causes have been excluded. It is advisable to conduct a comprehensive assessment of such patients using relevant laboratory tests, including liver and kidney function tests, blood cultures, and others, to identify key factors that may initially seem unlikely but are important for making the correct diagnosis.

## Data Availability

The original contributions presented in the study are included in the article/supplementary material, further inquiries can be directed to the corresponding authors.
